# Genetic variation of CXCR4 and risk of coronary artery disease: epidemiological study and functional validation of CRISPR/Cas9 system

**DOI:** 10.18632/oncotarget.23491

**Published:** 2017-12-15

**Authors:** Guo Runmin, Jiang Jiamei, Jing Zhiliang, Chen Yonghua, Shi Zhizhou, Tao Guizhou, Liu Shuguang

**Affiliations:** ^1^ Department of Cardiology, Affiliated Hospital of Guangdong Medical University, Zhanjiang, Guangdong 524001, P.R. China; ^2^ Department of Pathology, Affiliated Hospital of Guangdong Medical University, Zhanjiang, Guangdong 524001, P.R. China; ^3^ Department of Endocrinology and Metabolism, Longgang District People's Hospital, Shenzhen 518172, China; ^4^ The Third People's Hospital of Dalian, Dalian, Liaoning 116091, P.R. China; ^5^ Department of Pathology, The Eighth Affiliated Hospital, Sun Yat-Sen University, Shenzhen, Guangdong 518033, P.R. China

**Keywords:** coronary artery disease, variation, genetic, CXCR4

## Abstract

Cardiovascular diseases (CVDs) remain the leading cause of death worldwide, while coronary artery disease (CAD) account for a large part of CVDs. Vascular CXCR4 could limit atherosclerosis by maintaining arterial integrity. Here, we conducted a population-based, case-control study to evaluate the associations of common genetic variation within the CXCR4 gene (rs2228014, rs117600832, rs2471859, and rs2322864) with CAD risk in a Chinese population. We found that CXCR4 rs2228014 was significantly associated with 1.29-fold increased risk of CAD (A vs G: OR = 1.29; 95% CI = 1.07–1.55; *P* = 0.007). The subjects with genotype AA (OR = 1.98; 95% CI = 1.03–3.81; *P* = 0.041) and AG (OR = 1.27; 95% CI = 1.02–1.58; *P* = 0.030) have higher risk of CAD, compared with those with genotype GG. Furthermore, both in the CAD patients with diabetes and those without diabetes, rs2228014 was significantly associated with increased risk of CAD (*P* < 0.05). Additionally, we also validated the significant association for rs2322864 (C vs T: OR = 1.20; 95% CI = 1.00–1.44; *P* = 0.046). Knockout of CXCR4 gene could significantly impair the capacity of cholesterol efflux (*P* < 0.01). These findings strongly suggest that CXCR4 polymorphisms might contribute to CAD susceptibility, and the exact biological mechanism awaits further research.

## INTRODUCTION

Coronary artery disease (CAD), also known as ischemic heart disease (IHD) and coronary heart disease (CHD), was the leading cause of death globally [1]. Although CAD mortality has gradually declined over the last decades in western countries, it still causes about one-third of all deaths in people older than 35 years worldwide [2]. Although traditional risk factors, such as age, male gender, smoking, alcohol drinking, obesity, hypertension, hypercholesterolemia, and diabetes mellitus have been identified to contribute to the pathogenesis of CAD, genetic factor also accounted for 30%~60% of the risk of CAD [3–6]. Genome-wide association studies (GWASs) have identified many susceptibility loci for CAD, although large part of genetic etiology of CAD could not be explained until now [7–10]. These also included the contribution of the famous Framingham Heart Study, which was established in 1948 by the US Public Health Service [11].

Recently, Doring et al [12] reported that vascular CXCR4 could limit atherosclerosis through maintaining arterial integrity, preserving endothelial barrier function. This means enhancing arterial CXCR4 might open novel therapeutic options in atherosclerosis. CXCR4-deficiency favored a synthetic phenotype, the occurrence of macrophage-like SMCs in the lesions, and impaired cholesterol efflux [12]. The CXCL12/CXCR4 axis have been found to play a critical role in coronary artery development [13]. A nested case-control study found that increased CXCR4 level in peripheral CD34+ cells was associated with good coronary collateralization in patients with chronic total coronary occlusion [14]. Genetic variations of CXCR4 have been linked to susceptibility of many phenotypes, including HIV/SIV infection, multiple cancers, and so on [15–18]. In this study, we performed a candidate gene study of CAD using a tag single-nucleotide polymorphism (tagSNP) approach for interrogating common genetic variation within the CXCR4 gene in a Chinese population.

## RESULTS

### Characteristics of study subjects

The characteristics of the study participants were summarized in Table 1. We totally included 1,200 unrelated CAD patients and 1,200 geographical-matched healthy controls in this study. No significant differences were found between cases and controls for age, gender, hypertension, and alcohol status. However, significant associations were detected for smoking status, family history of CAD, diabetes status, body-mass index, TC, TG and HDL-C (*P* < 0.005). When included in a logistic regression model, smoking status, family history of CAD, diabetes status, TC, TG and HDL-C were determine as the risk predictors of CAD.

**Table 1:** Clinical demographic characteristics of CAD cases and controls

Variables	Cases (*n* = 1200)	Controls (*n* = 1200)	*P* value
Age			
≥ 60	564 (47.0%)	596 (49.7%)	0.191
< 60	636 (53.0%)	604 (50.3%)	
Gender			
Male	877 (73.1%)	859 (71.6%)	0.411
female	323 (26.9%)	341 (28.4%)	
Smoking status			
Smokers	422 (35.2%)	241 (20.1%)	***P* < 0.001**
Non-Smokers	778 (64.8%)	959 (79.9%)	
Alcohol status			
drinkers	269 (22.4%)	250 (20.8%)	0.346
Non-drinkers	931 (67.6%)	950 (79.2%)	
Diabetes			
Yes	449 (37.4%)	97 (8.1%)	***P* < 0.001**
No	751 (62.6%)	1103 (91.9%)	
Hypertension			
Yes	412 (34.3%)	433 (36.1%)	0.369
No	788 (65.7%)	767 (63.9%)	
Family history of CAD			
Yes	127 (10.6%)	302 (25.2%)	***P* < 0.001**
No	1073 (89.4%)	898 (74.8%)	
Body-mass index	24.1 ± 2.4	23.9 ± 2.2	**0.033**
TC (mmol/L)	4.55 ± 0.65	4.09 ± 0.55	***P* < 0.001**
TG (mmol/L)	1.68 ± 0.19	1.58 ± 0.18	***P* < 0.001**
HDL-C (mmol/L)	1.55 ± 0.26	1.44 ± 0.24	***P* < 0.001**

### Genotype analyses

Table 2 summarized the genotypic frequencies of the four tagSNPs of the CXCR4 gene (rs2228014, rs117600832, rs2471859, and rs2322864) in Chinese CAD patients and healthy controls. The distribution of all the four CXCR4 variants in the controls was compatible with HWE (*P* > 0.05). After adjusted for age, gender, smoking status, drinking status, diabetes, hypertension, smoking status, family history of CAD, body-mass index, TC, TG and HDL-C, SNP rs2228014 showed significant association with increased CAD risk (A vs G: OR = 1.29; 95% CI = 1.07–1.55; *P* = 0.007). The subjects with genotype AA (OR = 1.98; 95% CI = 1.03–3.81; *P* = 0.041) and AG (OR = 1.27; 95% CI = 1.02–1.58; *P* = 0.030) have higher risk of CAD, compared with those with genotype GG. For rs2322864, we also detected a significant association (C vs T: OR = 1.20; 95% CI = 1.00–1.44; *P* = 0.046). No significant association was detected for CXCR4 rs117600832 and rs2471859.

**Table 2:** Association between genetic variations of CXCR4 and risk of CAD

	CAD cases	Controls	OR (95% CIs)^*^	*P* value
rs2228014				
GG	844	896	1.00 (Reference)	
AG	331	290	1.27 (1.02–1.58)	**0.030**
AA	25	14	1.98 (1.03–3.81)	**0.041**
A vs G			1.29 (1.07–1.55)	**0.007**
AA+AG vs GG	356/844	304/896	1.29 (1.06–1.58)	**0.013**
AA vs AG+GG	25/1175	14/1186	1.87 (0.97–3.63)	0.063
rs117600832				
GG	1036	1045	1.00 (Reference)	
CG	151	145	1.09 (0.71–1.67)	0.691
CC	13	10	1.35 (0.55–3.30)	0.511
C vs G			1.11 (0.82–1.49)	0.492
CC+CG vs GG	164/1036	155/1045	1.11 (0.77–1.59)	0.570
CC vs CG+GG	13/1187	10/1190	1.35 (0.55–3.35)	0.510
rs2471859				
AA	1049	1051	1.00 (Reference)	
AG	139	140	1.03 (0.61–1.73)	0.911
GG	12	9	1.38 (0.54–3.53)	0.501
G vs A			1.07 (0.84–1.38)	0.600
GG+AG vs AA	151/1049	149/1051	1.06 (0.46–2.40)	0.897
GG vs AA+AG	12/1188	9/1191	1.39 (0.54–3.54)	0.490
rs2322864				
TT	789	830	1.00 (Reference)	
CT	376	341	1.21 (0.99–1.48)	0.061
CC	35	29	1.31 (0.80–2.15)	0.288
C vs T			1.20 (1.00–1.44)	**0.046**
CC+CT vs TT	411/789	370/830	1.22 (1.01–1.47)	**0.040**
CC vs CT+TT	35/1165	29/1171	1.30 (0.78–2.16)	0.312

^*^ Adjusted for age, gender, smoking status, drinking status, diabetes, hypertension, smoking status, family history of CAD, body-mass index, TC, TG and HDL-C.

### Stratified analyses

To further evaluate the potential effect modification of the diabetes status, stratified analyses were conducted for rs2228014 (Table 3). Both in the CAD patients with diabetes and those without diabetes, rs2228014 was significantly associated with increased risk of CAD, which confirmed the robustness of the findings.

**Table 3:** Association between CXCR4 rs2228014 and Risk of CAD stratified by diabetes

	CAD cases	Controls	OR (95% CIs) ^*^	*P* value
CAD with diabetes				
GG	315	896	1.00 (Reference)	
AG	124	290	1.27 (0.96–1.68)	0.095
AA	10	14	2.12 (0.97–4.65)	0.061
A vs G			1.31 (1.03–1.66)	0.026
AA+AG vs GG	134/315	304/896	1.30 (1.00–1.69)	0.049
AA vs AG+GG	10/339	14/1186	2.60 (1.18–5.72)	0.018
CAD without diabetes				
GG	529	896	1.00 (Reference)	
AG	207	290	1.27 (0.99–1.62)	0.055
AA	15	14	1.89 (0.91–3.93)	0.088
A vs G			1.29 (1.05–1.59)	0.017
AA+AG vs GG	222/529	304/896	1.29 (1.02–1.62)	0.032
AA vs AG+GG	15/736	14/1186	1.79 (0.85–3.77)	0.122

^*^ Adjusted for age, gender, smoking status, drinking status, hypertension, smoking status, family history of CAD, body-mass index, TC, TG and HDL-C.

### Cholesterol efflux evaluation

**Figure 1:**
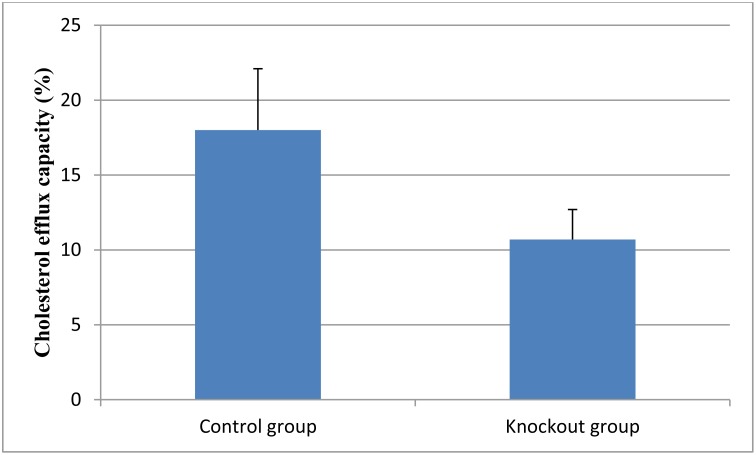
Cholesterol efflux capacity in the knockout and control group Data are expressed as mean ± SD.

Furthermore, we evaluated the effect of knockout of the CXCR4 gene on the lesional cholesterol efflux. As shown in Figure 1, cholesterol efflux capacity was significantly lower in the CXCR4 knockout group (*P* < 0.01).

## DISCUSSION

The current study explored the associations between four genetic variants of the CXCR4 gene and risk of CAD in a population-based, case-control study. We found that CXCR4 rs2228014 was significantly associated with 1.29-fold increased risk of CAD. Furthermore, both in the CAD patients with diabetes and those without diabetes, rs2228014 was significantly associated with increased risk of CAD. We also validated the significant association for rs2322864 in CAD patients.

CXCR4 gene was located at 2q22.1. This gene encodes a CXC chemokine receptor specific for stromal cell-derived factor-1, while the encoded protein has 7 transmembrane regions and is located on the cell surface [19]. CXCR4 was first found to mediate CD4-independent infection by HIV-2 in 1996 [20]. Since, there is increasing evidence that abnormal expression and genetic variation of CXCR4 is involved in several pathological conditions, including immune diseases, viral infections, multiple cancers, and cardiovascular diseases [21–35]. Recently, vascular CXCR4 was identified to limits atherosclerosis by maintaining arterial integrity, and rs2322864, a SNP located within the CXCR4 locus, was detected to be associated with increased risk for CAD [12]. The mechanism referred that CXCL12/CXCR4 chemokine ligand/receptor axis promoted endothelial barrier function through VE-cadherin expression and the stabilization of junctional VE-cadherin complexes [12].

With this background, we evaluated the genetic variants of the CXCR4 gene with risk of CAD, which identified a significant association for rs2228014 and validated the significant association for rs2322864 in CAD patients. Combinational polymorphisms of seven CXCL12-related genes, including rs2228014, were found to be protective against breast cancer in Taiwan first [36]. A meta-analysis including 3684 cancer patients and 5114 healthy controls participating in 11 studies also showed that rs2228014 polymorphism was associated with a significantly increased risk of cancer in homozygote model (OR = 2.01, 95% CI: 1.22–3.33) and in recessive model (OR = 1.97, 95% CI: 1.23–3.16) [37]. In current study, our results indicated that SNP rs2228014 showed significant association with increased CAD risk (A vs G: OR = 1.28; 95% CI = 1.07–1.54; *P* = 0.007).

This study also has several strength. First, the genetic background of cases and controls was well matched. To avoid false-positive associations caused by differences in age, gender and other covariates, we selected cases and controls that are well matched for age and gender, and ensured that the associations remained significant after adjustment for potential confounding bias; Second, the sample size were adequate; Third, results of rs2228014 remained significant after Bonferroni correction for multiple comparisons (0.007*4 = 0.028, Bonferroni-adjusted). Also, several limitations of our study should be considered. First, inherent selection bias for case-control study; second, lack of replication in an independent population; third, we didn't reach a Bonferroni-adjusted statistical significance for rs2322864, although our sample size was moderate for a common variant; fourth, some other confounding factors may potentially mediate the effect of selected polymorphisms on CAD risk such as physical activity, diet habit, family history of other cardiovascular diseases and so on. However, these limitations do not detract from the main conclusions.

In conclusion, our study provided evidence that CXCR4 rs2228014 and rs2322864 were significantly associated with increased risk of CAD. The replications of our studies in other populations as well as further systematic investigations are needed to clarify the molecular mechanisms underlying CXCR4 regulation.

## MATERIALS AND METHODS

### Study subjects

In current study, we included a total of 1,200 unrelated patients with CAD which were recruited between before Oct 2016. The diagnosis of CAD was certified by coronary angiography performed via a quantitative coronary angiographic system. CAD was defined as luminal narrowing of more than 50% in one or more main coronary arteries. Control subjects comprised 1,200 healthy subjects from the same geographical area who were undergoing a routine check-up. All the participants were Chinese-Han population. A face to face interview and a review of the medical records were implemented to collect the demographic and clinical characteristics data. After the interview, five milliliter peripheral venous blood was collected in tubes containing disodium-EDTA as an anticoagulant and then stored at −80^°^C until genomic DNA extraction. All the subjects included in this case-control study were given an informed consent and also the study protocol.

### Variable definition

Diabetes was diagnosed with at least one of the following criteria: 1) a random venous plasma glucose concentration ≥ 11.1 mmol/l; 2) a fasting plasma glucose concentration ≥ 7.0 mmol/l; 3) two hour plasma glucose concentration ≥ 11.1 mmol/l (two hours afer 75 g anhydrous glucose in an oral glucose tolerance test). A diagnosis of hypertension was based on the presence of elevated systolic (≥ 140 mmHg) and/or diastolic (≥ 90 mmHg) blood pressure, or current use of antihypertensive medications. Smoking was defined as the non-casual current or ever inhalation of the smoke of burning tobacco encased in cigarettes, pipes, and cigars. While alcohol status was defined as frequent alcohol consumption.

### TagSNP selection, DNA extraction, and genotyping

The TagSNPs were selected using the pairwise LD function of the SNAP (https://www.broadinstitute.org/mpg/snap/) web server [38]. These resulted three tagSNPs of the CXCR4 gene were selected, including rs2228014, rs117600832, and rs2471859. We also including the SNP rs2322864, which was located in the flanking region of the CXCR4 gene and detected previously [12]. Genomic DNA used for SNPs genotyping was extracted from peripheral blood lymphocytes using a DNA extraction kit (TianGen, Beijing, China). The genotyping was conducted using SEQUENOM Mass-ARRAY system. For quality control, genotyping was performed without knowledge of the case or control status. One hundred random-selected samples were tested in duplicate, and the reproducibility was 100%.

### CRISPR/Cas9-mediated knockout of the CXCR4 gene

The guide RNAs were designed to recognize the CXCR4 Gene using the CRISPR Design Tool (http://crispr.mit.edu/). The guide RNA with the highest score was selected, and cloned into the PGL3 plasmid. Precise genome editing in mice was performed using the 3-component CRISRP-Cas9 system, while the knockout of CXCR4 gene was confirmed by sequencing. Then we analyzed the effect of CRISPR/Cas9-mediated knockout of the CXCR4 gene on the lesional cholesterol efflux. Efflux is given as the percentage of counts recovered from the medium in relation to the total counts present on the plate. All efflux experiments were performed in duplicate for each sample.

### Statistical analysis

The statistical analysis on the characteristics of the subjects was performed with Student's *t*-test for the continuous variables, while Pearson x2 test was used for the categorical variables. The genotypes were tested for Hardy–Weinberg equilibrium (HWE) using Fisher's exact test in controls. The logistic regression models were performed to calculate the odds ratios (ORs) and 95% confidence intervals (CIs) and adjust the potential confounding factors by including these factors in the regression models. Statistical analysis was performed on SPSS v. 19.0 software (SPSS, Chicago, IL). A *P* value < 0.05 was considered statistically significant.
